# Role of UV radiation and oxidation on polyethylene micro- and nanoplastics: impacts on cadmium sorption, bioaccumulation, and toxicity in fish intestinal cells

**DOI:** 10.1007/s11356-024-34301-x

**Published:** 2024-07-17

**Authors:** Estefanía Pereira Pinto, Justin Scott, Kendra Hess, Estefanía Paredes, Juan Bellas, Jorge Gonzalez-Estrella, Matteo Minghetti

**Affiliations:** 1 Department of Integrative Biology, Oklahoma State University, Stillwater, OK 74078 USA; 2 Centro de Investigación Mariña, Departamento de Ecoloxía e Bioloxía Animal, Laboratorio de Ecoloxía Costeira (ECOCOST), 36310, Universidade de Vigo, Vigo, Spain; 3 School of Civil and Environmental Engineering, Oklahoma State University, Stillwater, OK 74078 USA; 4 Centro Oceanográfico de Vigo, Instituto Español de Oceanografía (IEO, CSIC), Subida a Radio Faro 50, 36390 Vigo, Spain

**Keywords:** Microplastic, Nanoplastic, Weathering, UV aging, Cadmium, Cell line

## Abstract

This study investigated the role of ultraviolet (UV) radiation and oxidation in high-density polyethylene microplastics (2–15 μm) and nanoplastics (0.2–9.9 μm) (NMPs) on particle chemistry, morphology, and reactivity with cadmium (Cd). Additionally, toxicity of NMPs alone and with Cd was evaluated using RTgutGC cells, a model of the rainbow trout (*Oncorhynchus mykiss*) intestine. The role on NMPs on Cd bioaccumulation in RTgutGC cells was also evaluated. Dynamic light scattering indicated that after UV radiation NPs agglomerated size increased from 0.8 to 28 µm, and to 8 µm when Cd was added. Oxidized MPs agglomerated size increased from 11 and 7 to 46 and 27 µm in non-UV- and UV-aged oxidized MPs when adding Cd, respectively. Cd-coated particles exhibited generally significantly higher zeta potential than non-Cd-coated particles, while attenuated total reflectance–Fourier transform infrared spectroscopy showed that the functional chemistry of the particles was oxidized and modified after being exposed to UV radiation. Presence of NMPs resulted in a significant decrease in Cd bioaccumulation in RTgutGC cells (100.5–87.9 ng Cd/mg protein) compared to Cd alone (138.1 ng Cd/mg protein), although this was not quite significant for co-exposures with UV-aged NPs (105.7 ng Cd/mg protein). No toxicity was observed in RTgutGC cells exposed to NMPs alone for 24 h. Moreover, co-exposures with Cd indicated that NMPs reduce the toxicity of Cd. Altogether these results show that UV aging enhances NMP surface reactivity, increasing Cd absorption in solution, which resulted in a reduction in Cd bioavailability and toxicity.

**Supplementary Information:**

The online version contains supplementary material available at 10.1007/s11356-024-34301-x.

## Introduction

Microplastics (MPs, 1 µm–5 mm) and nanoplastics (NPs, < 1 µm) (NMPs) are ubiquitous in the environment and are increasingly becoming of concern based on their prevalence, large production volume, environmental interactions, and limited toxicological information (Leusch and Ziajahromi 2021). Concentrations in the aquatic environment vary, with reported values in highly polluted areas of 1.26 mg/L in the Saigon River of Vietnam (Strady et al. 2020; Xiong et al. 2023), 4.5 mg/L in the Southern Sea of Korea (Kang et al. 2015; Green et al. 2017), or 4.65 mg/L in the Yellow River estuary of China (Han et al. 2020; Xiong et al. 2023). Notably, there is a significant lack of data on particle concentrations below 300 μm and their weathering status (Almeida et al. 2019; Oliveira et al. 2019), raising concerns about the accuracy of reported environmental concentrations. Additionally, although some environmental solutions for plastic waste have been already proposed, such as recycling by pyrolysis or its conversion to char composite materials for producing magnetic char composites (Osman et al. 2020, 2022), with the ongoing unregulated release and degradation of plastics, NMP concentrations are expected to increase steadily in the future (Koelmans et al. 2016; Garcia-Muñoz et al. 2023).

Fish represent a relevant target of NMPs, as many species have been reported to ingest these particles worldwide (Wootton et al. 2021). Micro- and NP ingestion can induce negative impacts on fish health and survival, leading to cascading impacts on the ecosystem (Barría et al. 2020; Benson et al. 2022) For example, effects reported on the freshwater species zebrafish (*Danio rerio*) include oxidative stress, inflammation, and lipid accumulation in liver after 7 days of exposure to 20–2000 µg/L of 5-μm and 70-nm polystyrene (PS) NMPs (Lu et al. 2016) or decreased length and heart rates attributed to < 230-μm polypropylene and < 100-μm PS plain and weathered NMP exposure for 4 days in concentrations ranging between 2000 and 200,000 particles/L and 12.5 and 100 mg/L (Prata et al. 2022). Previous findings in the marine species juvenile grouper *Epinephelus moara* indicated disruption of the hepatic lipid homeostasis after exposure to 2 and 20 mg/g of 20- to 100-μm weathered and plain PS MPs for 26 days (Wang et al. 2020c).

Moreover, NMP ability to act as vectors of organic and inorganic pollutants and to modulate the bioaccumulation and toxicity of these contaminants has been highlighted in previous studies (Koelmans et al. 2016; Chen et al. 2017; Pannetier et al. 2019; Hu et al. 2022). Among these pollutants, cadmium (Cd) is a nonessential metal which poses toxicity for aquatic organisms even at low concentrations (Cicik and Engin 2005), but the levels at which it causes acute and chronic toxicity can vary greatly mostly depending on water hardness and presence of dissolved organic matter (Niyogi et al. 2008), but freshwater fish and invertebrates are among the most sensitive to its effects (Wright and Welbourn 1994). After 4 days of exposure to 2.0–2.8 mg Cd/L, rare minnows (*Gobiocypris rarus*) experienced necrosis of hepatocytes, accumulation and abnormal deposition of cytoplasmic lipid droplets, formation of nuclear lipid droplets, and abnormal increases in rough endoplasmic reticulum (Liu et al. 2023). Additionally, crucian carp, *Carassius auratus gibelio*, showed damage in intestinal structure after 4 weeks of 100–500 μg Cd/L, alongside elevation of intestinal apoptosis enzymes and apoptosis rate of enterocytes at high Cd concentrations (Yu et al. 2021). At the cellular level, in rainbow trout intestinal cells, it was shown that 4158 μg/L induces a 50% reduction in cell viability after 24 h of exposure and that it induces expression of metallothionein and glutathione reductase, indicating oxidative stress (Oldham et al. 2023). Cadmium is easily encountered in the environment due to its wide use in batteries, metallic alloys, pigments, and plastic stabilizers, among others with concentrations ranging between 0.001 and 1.25 ppb (Pan et al. 2010; Yu et al. 2010; Wang et al. 2019; Sadiq 2021). It can be found in freshwater and marine environments as positively charged ions (e.g., Cd^2+^ and CdCl^+^), which facilitates the adsorption to the negatively charged surface of plastic particles in the environment (Holmes et al. 2012; Sadiq 2021; Quiambao et al. 2023). Accordingly, previous studies found that co-exposure to polyethylene (PE) MPs increased Cd toxicity in common carp (*Cyprinus carpio*) at 0.25–0.5 mg MPs/L by means of biomarkers of liver function and metabolic stress, while it decreased toxicity in goby (*Pomatoschistus microps*) at 0.012–1.5 mg MPs/L of 1–5 µm PE NMPsin terms of predatory performance and acetylcholinesterase activity (Banaee et al. 2019; Miranda et al. 2019).

Nevertheless, limited information is available about the role of environmental weathering on MPs’ and NPs’ sorption of contaminants (Liu et al. 2020; Wang et al. 2020a) and possible adverse effects on organisms (Di Natale et al. 2022; Miranda et al. 2022). Micro- and NPs are exposed to weathering conditions in nature including mechanical abrasion, chemical, biological and thermal degradation, and photodegradation, which may affect their surface and bulk chemistry (Yousif and Haddad 2013; Al-Mashhadani et al. 2021; Zhang et al. 2021). Photodegradation of plastics is mainly caused by photons in the range of the infrared to ultraviolet (UV) spectrum (Wayman and Niemann 2021). The degradation of plastic particles under UV light in air, freshwater, and seawater was simulated in previous research (Cai et al. 2018; Mao et al. 2020; Pinlova and Nowack 2024). Ultraviolet degradation results in yellowing, embrittlement, and a general decrease in the mechanical properties of most polymers (Liu et al. 2009; Wang et al. 2021a; Belone et al. 2022). Ultraviolet radiation induces chemical reactions that expose polymers to hydroxide (OH^−^) and hydroperoxyl (HO_2_) radicals, modifies surface charge, and releases plastic impurities such as trace metals, additives, or solvents (Yousif and Haddad 2013; Amelia et al. 2021; Di Natale et al. 2022; El Hayek et al. 2023). In addition to artificial photoaging, advanced oxidation processes simulate natural aging, leading to rough, cracked surfaces and fragmentation (Liu et al. 2019; Hu et al. 2023). The physicochemical alterations caused in NMPs by weathering can affect the interaction of particles with environmental co-existing pollutants, for instance increasing the adsorption of heavy metals and modulating pollutants’ inherent toxicity (Mao et al. 2020; Wang et al. 2021b).

In this research, we aimed to investigate (1) the role of surface chemical modifications (plain vs. oxidized MPs), photodegradation (UV aged vs. non-UV aged) and size (MPs vs. NPs) on plastic particles ability to sorb Cd. We used high-density polyethylene (HDPE) NMPs, which constitute the most produced polymer and the most commonly found in the aquatic environment (Andrady 2011; Besson et al. 2020). Moreover, we intended to evaluate how surface chemistry, photodegradation, and size related to (2) the toxicity of HDPE NMPs in presence or absence of Cd in cells derived from the rainbow trout (*Oncorhynchus mykiss*) intestine (RTgutGC) and (3) the bioaccumulation of Cd on these cells in presence or absence of NMPs. Therefore, the main two hypotheses of this study were that the particles alone are capable of causing toxicity to RTgutGC cells, and second, that MPs are capable of acting as carriers of metals (Cd), modulating their toxicity and bioaccumulation (Minghetti et al. 2017). RTgutGC cells have been shown to form a polarized epithelium comprising tight junction proteins, which may prevent the entry of exogenous colloidal contaminants paracellularly, and it has been previously employed to determine nanoparticle toxicity and bioaccumulation (Minghetti and Schirmer^ 2016^; Geppert et al. 2021). Also, it is physiologically relevant to study NMP bioaccumulation and toxicity in fish focusing on the dietary routes, especially in marine fish that drink water for osmoregulatory purposes (Tytler et al. 1990; Jovanović et al. 2018; Pannetier et al. 2020; Sales-Ribeiro et al. 2020). In order to comprehensively assess the potential impacts of NMPs on aquatic organisms, we established the test concentrations within a wide range (12.5 to 200 mg/L). This decision is based on the in vitro model system and the exposure period selected (24 h), and constitutes a common methodology adopted across numerous laboratory studies assessing the toxicity of NMPs, where mg/L concentrations serve as exposure thresholds to enhance sensitivity in detecting toxic effects and explore mechanisms of action (Hoang and Felix-Kim 2020; Jakubowska et al. 2022; Yu et al. 2022; Lee et al. 2023; Wang et al. 2023). Moreover, while the chosen concentrations slightly exceed the upper limits observed in field surveys conducted in various aquatic environments (Kang et al. 2015; Green et al. 2017; Han et al. 2020; Strady et al. 2020; Xiong et al. 2023), a rise in the concentrations of NMPS is expected in the future as a result of uncontrolled discharge and degradation of plastic in the nature (Koelmans et al. 2016; Garcia-Muñoz et al. 2023).

Our study provides novel information about the role of particle size, surface and functional chemistry, and morphology of NMPs on sorption to Cd on their toxicity alone or with Cd and bioaccumulation of Cd in fish intestinal cells. This research is the first to elucidate the role on NMP oxidation and UV radiation on their surface reactivity with Cd and how specific properties of NMPs affect the modulation of Cd toxicity and bioaccumulation in RTgutGC cells. Our findings enhance the potential effects of NMPs on modifying metal uptake dynamics and toxicity in aquatic ecosystems.

## Materials and methods

### Plastic *particles and weathering process*

One batch of plain and oxidized HDPE MPs were purchased from Micropowders Inc. (Tarrytown, NY, USA), while one batch of NPs was acquired from Cospheric (Santa Barbara, CA, USA). The NMP characterization details sourced from the manufacturers are provided in the supporting information (SI) file (Text S1).

The three particles’ groups were exposed to UV light (302 nm) for 42 days in open glass Petri dishes placed 30 cm away from the light source in a Bio-Rad Gel Doc XR + Gel Documentation System (Bio-Rad, Hercules, CA, USA) in the presence of oxygen. This simulated environmental photooxidation weathering, following prior research (Wang et al. 2020a; Cheng et al. 2021; Liu et al. 2021; Ouyang et al. 2022). Daily manual shaking under a lidded arrangement ensured even UV aging for experimental analysis and exposures, preventing cross-contamination. This weathering process generated the UV-aged MPs, UV-aged oxidized MPs, and UV-aged NPs employed in the exposure experiments.

### Exposure medium preparation

This study utilized L-15/ex as the exposure medium for RTgutGC cell line cultures. It is a synthetic medium identical to Leibovitz’s L-15 medium (Thermo Scientific, Waltham, MA, USA), but devoid of amino acids and vitamins to prevent metal chelation (Minghetti and Schirmer 2016). This medium presents an osmolarity of ~ 300 mOsm/L and a composition comparable to fish intestinal lumen in freshwater (Shehadeh and Gordon 1969; Ibrahim et al. 2020). Exposure solutions were freshly prepared by weighing the mass of plastic needed in a precision balance (Mettler Toledo XP2U, Brooklyn, NY, USA) and suspending the plastics in L-15/ex in polypropylene Falcon tubes. Exposure solutions were sonicated for 20 min in an ultrasonic water bath prior to exposure or measurement, to ensure homogeneity of the suspension. Concentrations of particles within the mg/L range (12.5–200 mg/L) were chosen on the basis of maximum reported concentrations found in the natural environment and previous toxicological studies (Kang et al. 2015; Green et al. 2017; Han et al. 2020; Hoang and Felix-Kim 2020; Strady et al. 2020; Jakubowska et al. 2022; Yu et al. 2022; Lee et al. 2023; Xiong et al. 2023). When Cd co-exposure experiments were run, an additional step was added, spiking 450 µg/L of Cd to the NMP solutions and allowing the adsorption of Cd for 48 h by mixing at 400 rpm in an orbital shaker. An 1830 mg/L stock solution of CdCl_2_ (Sigma-Aldrich, St. Louis, MO, USA) in ultrapure water (16–18 mΩ, Barnstead GenPure Water, Thermo Fisher Scientific, Waltham, MA, USA) was prepared in a polypropylene Falcon tube and diluted in L-15/ex to achieve the desired 450 µg/L Cd concentration in the exposure media. The rationale for selecting this concentration is based on its detectability through ICP-OES, together with its demonstrated ability to cause an approximately 10% reduction in RTgutGC cell viability (Oldham et al. 2023). Cadmium concentration in the stock and exposure solutions was measured by ICP-OES as previously described (Oldham et al. 2023). Standard reference material (NIST SRM 1643f; National Institute of Standards and Technology, MD, USA) was also analyzed to ensure the quality of the measurement. All measured concentrations were within 5% of nominal values (Fig. S1, Supporting Information).

### Characterization of the NMPs

#### Dynamic light scattering and zeta potential

Dynamic light scattering (DLS) and zeta potential were performed to define the size and surface electrical charge of the particle aggregates, and to determine the behavior of the particles in the media. Three particle suspensions were prepared following the same procedure as described in the “Exposure medium preparation” section. To conduct DLS measurements, concentrations of 10 mg MPs/L and 1 mg NPs/L of UV- and non-UV-aged particles were utilized, along with the introduction of 450 µg/L of Cd. Zeta potential determinations were performed for all particle types and treatments at a concentration of 25 mg plastic/L. Volumes of 1.5 mL and 50 µL were used to define particle size and zeta potential, respectively, using a ZetaPALS analyzer (Brookhaven Instruments Co., Holtsville, NY, USA), and a minimum of five replicate measurements were performed.

#### Scanning electron microscopy/energy dispersive X-ray spectroscopy

Scanning electron microscopy/energy dispersive X-ray spectroscopy (SEM/EDS) analyses were performed in a Thermo Fisher Scios2 dual beam SEM with Thermo Fisher Ultra Dry EDS system and Pathfinder software or Thermo Fisher FEI Quanta 600 FEG SEM with Bruker Quantax EDS system and Esprit software. These analyses assessed morphology of the particles and the possible changes occurred due to UV aging. Details of sample preparation are available in SI (Text S1).

#### Attenuated total reflectance–Fourier transformed infrared spectroscopy

Attenuated total reflectance–Fourier transformed infrared (ATR-FTIR) spectroscopy was performed in an iN10-MX micro-FTIR (Thermofisher, Waltham, MA, USA). This methodology was used to determine the chemical composition of the sample and detect differences in the plastic functional chemistry after the UV aging process and Cd addition. Details regarding the sample preparation and measurement parameters are provided in SI (Text S1).

To understand the effect of UV and chemical oxidation on the functional chemistry of NMPs, we calculated the carbonyl index (CI) according to Gomes et al. (2024). The CI was calculated as the area ratio between the absorbance of the carbonyl (C = O) peak (1850–1650 cm^−1^) and the methylene stretch peak (3020–2760 cm^−1^):

1 $$CI=\frac{{I}_{1850-1650}}{{I}_{3020-2760}}$$

#### Metal sorption to particles

Sorption experiments were run in triplicate. All particles were dispersed at 25 mg/L in L-15/ex containing 450 µg/L of Cd, as described in the “Exposure medium preparation” section. To evaluate the time needed to achieve the maximum adsorption of Cd to NMPs, the concentration of Cd was measured overtime for a period of 48 h. Samples of the liquid phase were collected at 0, 6, 24, and 48 h. Equilibrium was observed for all particles at 48 h (Fig. S2). Therefore, the results compared in this study are limited to samples collected at 0 and 48 h, capturing the most significant differences in sorption. Liquid samples (4 mL) were passed through a 0.22-µm polyethylene terephthalate (PET) filter. The PET filter was preserved for Cd desorption analysis. Cadmium concentration was measured by inductively coupled plasma optical emission spectrometry (ICP-OES, Thermo Fisher Scientific, Dreieich, Germany). Further details about the sampling methodology and reagent information are provided in SI (Text S1).

#### RTgutGC cell culture

Cells were routinely cultured as previously described (Minghetti and Schirmer 2016). To seed cells for exposure experiments, monolayers were washed twice with Versene (Thermo Fisher Scientific, Waltham, MA, USA) and detached using 0.25% Trypsin (Thermo Fisher Scientific, Waltham, MA, USA). Cell suspensions were counted on hemocytometer and viability was evaluated using Trypan Blue exclusion assay in a Countess II™ Automated Cell Counter (Life Technologies Corporation, NYC, NY, USA). Only cell batches with viability higher than 90% were used for seeding in bioaccumulation and cytotoxicity assays.

#### Cadmium bioaccumulation

Cells were seeded into 6-well plates (Greiner Bio-One, Monroe, NC, USA) at 80,000 cells/cm^2^, as described previously (Minghetti and Schirmer 2016). After 48 h of incubation at 19 °C, each confluent cell monolayer was washed twice with L-15/ex. Cells were then exposed to 450 µg/L of Cd in presence or absence of 25 mg/L of UV- and non-UV-aged NMPs mixed with 450 µg/L of Cd prepared as described in the “Exposure medium preparation” section. In this experiment Cd bioaccumulation in absence of NMPs was compared to Cd bioaccumulation in RTgutGC cells co-exposed to Cd-NMPs. The concentrations of Cd in the exposure solution were confirmed before and after exposure, via ICP-OES, as mentioned previously (Exposure medium preparation” section). After exposure, cells were washed, lysed, and digested as described previously (Minghetti and Schirmer 2016). One-tenth of the cell lysate volume was used for protein quantification using the Lowry Assay (Thermo Fisher Scientific, Waltham, MA, USA). This allowed to normalize differences in cell number in different wells. Cadmium accumulation data was presented as ng metal per mg of protein (Minghetti and Schirmer 2016).

#### Cell viability assays

A minimum of three independent replicates of the cytotoxicity assays were carried out with cells of different passages (71–76) in 24-well plates. RTgutGC cell monolayers were seeded similarly to the bioaccumulation experiment. A stock solution of 200 mg/L was prepared and serial dilutions were made to achieve the exposure solutions of NMPs from 12.5 to 200 mg/L (consistent with the concentrations displayed by other studies) (Wang et al. 2020b; Kim et al. 2021). Exposures were performed in presence or absence of 450 µg/L Cd. At 24 h post-exposure, the multiple cellular endpoint viability assay was performed as previously described (Minghetti and Schirmer 2016). All cytotoxicity experiments included a negative (exposure medium alone, L-15/ex) and a positive control, which consisted of 2700 µg/L of Cd solution dissolved in L-15/ex, corresponding to the EC_30_ value previously shown for metabolic activity (Oldham et al. 2023). Each experimental condition was present in three replicate wells and each experiment was repeated at least three times in 3 separate days with cells of different passages. Moreover, it should be noted that Cd was 100% soluble at all the concentration tested as determined by the chemical equilibrium model Visual MINTEQ (Oldham et al. 2023). In all experiments, the positive control showed an inhibition of 72% ± 10. Viability results are expressed as % cell viability in comparison to control (L-15/ex).

### Data analysis

Statistical analyses were performed using GraphPad Prism Version 9.0 (GraphPad Software Inc., San Diego, CA). All data were assessed for normality with the D’Agostino and Pearson normality test and homogeneity of variances was verified by using Levene’s test. All data were determined to follow a normal distribution, thus requiring no transformations. With the data confirmed as both normal and homogeneous, the analysis proceeded with analysis of variance (ANOVA) followed by Dunnett’s or Tukey’s post hoc tests to determine the statistical significance among different experimental groups (more than two groups). A Student’s *t*-test was performed to evaluate significant differences when only two groups were analyzed after checking for normality (D’Agostino and Pearson) and homogeneity of variances (Levene). The experimental design included a minimum of three replicates for all experiments. Statistical analyses were carried out using an alpha level of 0.05.

## Results and discussion

### Charge and size of NMPs in exposure media

**Fig. 1:**
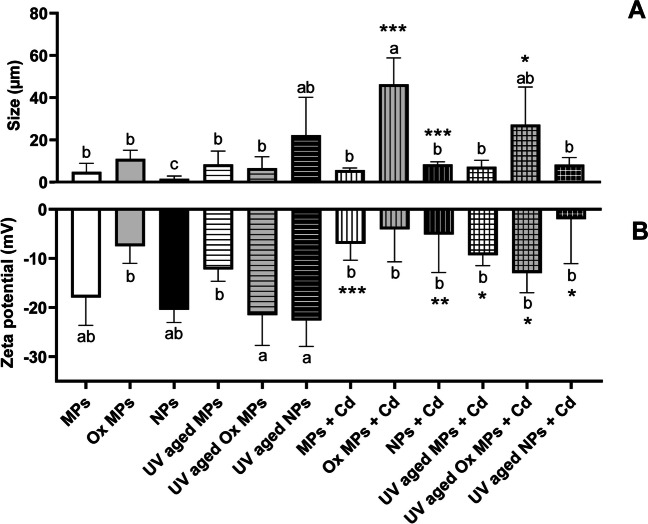
Dynamic light scattering (DLS) (**A**) and zeta potential (**B**) analysis of the high-density polyethylene microplastics and nanoplastics (HDPE NMPs) before and after UV and Cd treatment. Data are presented as mean ± SD. Lower case letters indicate significantly different effects among treatments and particle type (one-way ANOVA, Tukey’s multiple comparison test; alpha = 0.05; *n* = 3). An asterisk is used to indicate statistical difference from particles alone in comparison to respective particles with Cd (unpaired *t* tests, where *, **, and *** represent *p*-values < 0.05, 0.01, and 0.001, respectively)

According to the DLS measurements, the size of the oxidized particle agglomerates increased twofold with respect to particles in ultrapure water, indicating that they tend to aggregate when suspended in L-15/ex media (Fig. S3). Moreover, while MPs (4.884 ± 4.028 µm) and oxidized MPs (11.038 ± 4.034 µm) showed no significant differences among them and neither of them were significantly affected by the UV treatment (8.393 ± 6.302 µm MPs and 6.654 ± 5.370 µm Ox MPs), UV-aged NPs increased significantly in agglomerated size (from 0.749 ± 0.411 to 27.970 ± 14.386 µm; *p* value = 0.0105) in respect to non-UV-aged NPs, indicating that the effect of UV radiation after 42 days of UV exposure was particle size dependent (Fig. 1A). The effect of Cd on NMPs agglomerations is described in the “Metal sorption to particles” section.

Zeta potential is intrinsically related to the aggregation of the particles. Values higher than − 10 mV show an incipient stability of the particles, while at − 5 mV a rapid aggregation of the particles can be expected (ASTM 1982). In our experiments, the size of oxidized and UV-aged MPs decreased as the charge became more negative (from − 5 to ~  − 21 mV) (Fig. 1 B). Thus, the decrease in the size of MPs can be explained by an electrostatic mechanism. A decrease in size of the agglomerates can be expected as the repulsion charges become stronger. Interestingly, the charge of NPs remained about the same (− 20.47 to − 22.62 mV) which indicates that the charge was not affected by the UV aging process, and therefore, the increase in size is likely the result of the fusion of NPs due to heat of the UV radiation process. In agreement with this observation, zeta potential alterations after particles aging have been previously found (Fan et al. 2021; Meng et al. 2021; Sarkar et al. 2021; Bhagat et al. 2022).

### NMP surface chemistry analyses

#### Scanning electron microscope analyses

**Fig. 2:**
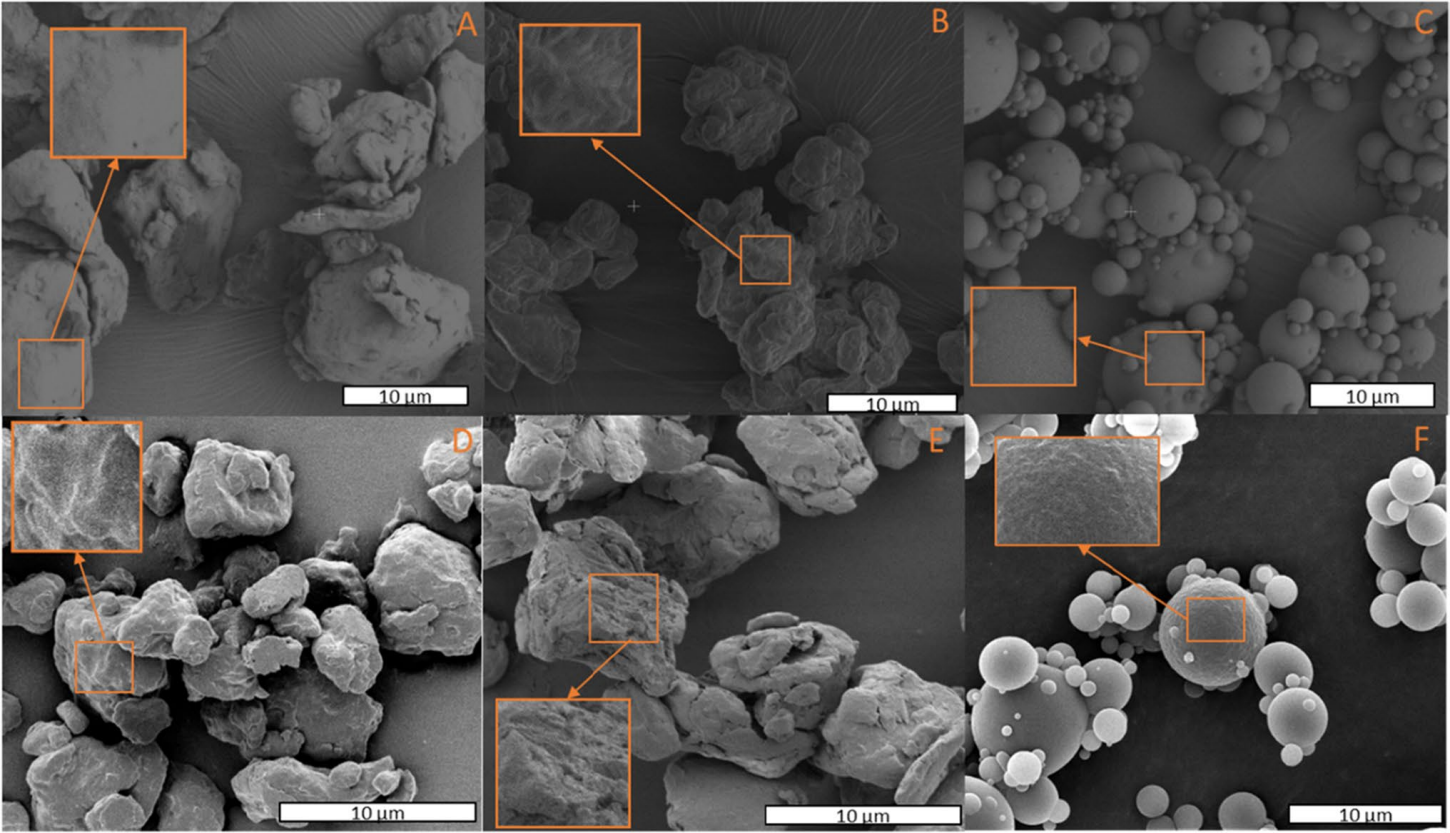
SEM images of HDPE NMPs. MPs (**A**); oxidized MPs (**B**); NPs (**C**); UV-aged MPs (**D**); UV-aged oxidized MPs (**E**); and, UV-aged NPs (**F**). Subpanels (× 3 to × 4 magnifications) inside each panel show changes in surface morphology due to oxidation and UV aging compared to non-aged MPs and NPs

SEM images revealed that the MPs’ surface was relatively smoother than that of the oxidized MPs (Fig. 2A and B), which presented a more rugged texture with pronounced cavities. Both MPs present sharp edges, while NPs are constituted by spheres of heterogeneous sizes and smooth surface (Fig. 2C). Literature data suggest that cracking and fragmentation may lead to a higher accessibility of light and oxygen to internal layers, producing a quicker aging of the particles (Liu et al.^ 2019^; Zhang et al. 2022). Analyses indicated that UV aging changed the texture and altered the surface morphology of MPs and NPs compared to the non-UV-aged particles (Fig. 2 D–F). This effect of photodegradation on the surface of plastic particles has been previously found (Andrady et al. 1998; Ma et al. 2019; Song et al. 2020; Sun et al. 2020; Alimi et al. 2022; Bond et al. 2022; El Hayek et al. 2023). Specifically, polymer’s absorption capability due to UV radiation can lead to photolytic, thermooxidative, and photooxidative reactions, with the latter reaction being recognized as the dominant factor in the weathering of plastics. Moreover, changes in texture, that lead to increase porosity, may enhance the surface area and sorption capacity of NMPs (Yu et al. 2019).

#### Attenuated total reflectance–Fourier transformed infrared spectroscopy of weathered particles

**Fig. 3:**
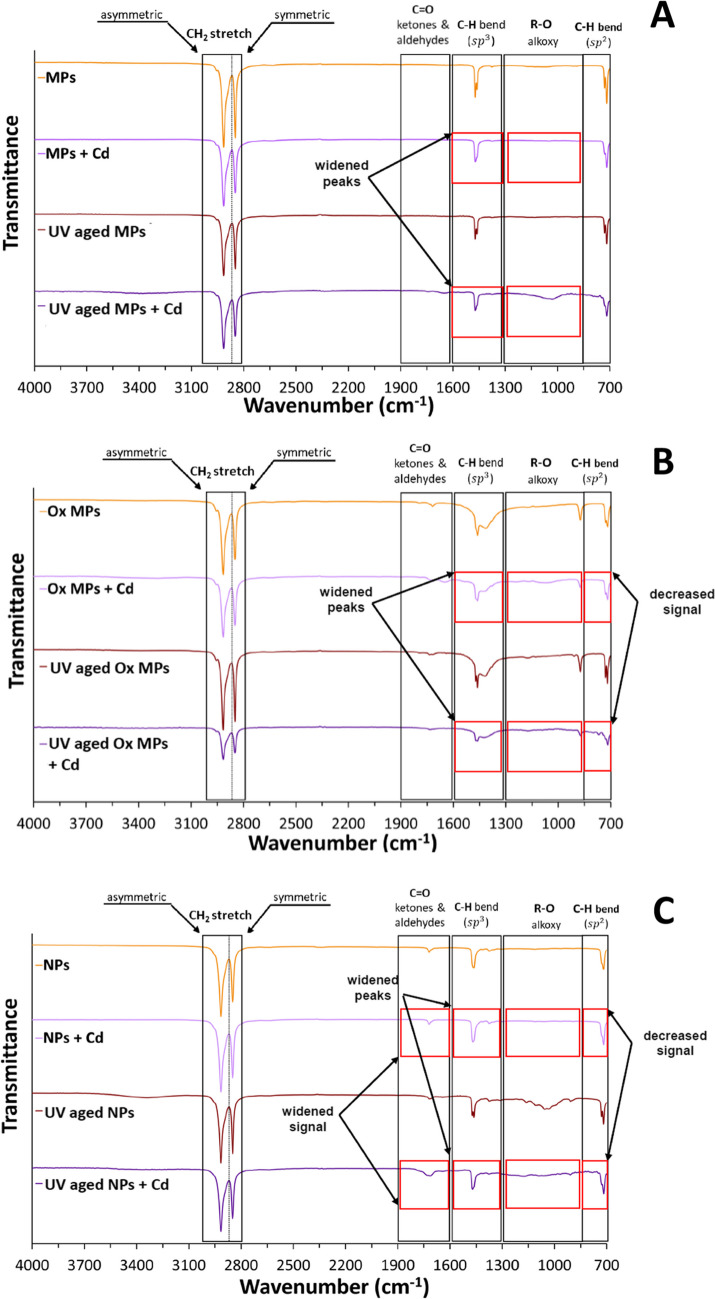
Attenuated total reflectance–Fourier transformed infrared spectroscopy (ATR-FTIR) spectra of UV- and non-UV-aged microplastics (MPs; **A**), oxidized microplastics (Ox MPs; **B**) and nanoplastics (NPs; **C**) before and after Cd adsorption

ATR-FTIR analyses confirmed that the acquired NMPs were high purity, high density polyethylene. Besides the morphological features, the chemical functional groups of MPs and NPs were also modified after being subjected to UV radiation aging. Figure 3 shows ATR-FTIR analyses of the NMPs under different conditions. While no effects were detected on MPs (Fig. 3A) after 6 weeks UV irradiation, these analyses demonstrated differences in the oxidized MP functional chemistry (Fig. 3B) determined by an increase in the C–H bending (1500–1300 cm^−1^ region) in the plastics subjected to UV radiation. The increased bend in the C–H bond observed in the 1000–800 cm^−1^ region may make the particles more susceptible to UV damage, which may suggest that oxidation causes the main effect on the functional chemistry of HDPE and enables further attack by UV irradiation (Tasumi 2014; Andrady 2015). Table S1 presents the CI for NMPs before and after UV aging. The data indicates that the purchased oxidized MPs and NPs already had some natural oxidation, and the CI of all NMPs increased after UV radiation.

Moreover, ATR-FTIR analyses of the UV-aged NPs indicated a change in the 3550–3250 cm^−1^ region commonly associated with hydroxyl groups (Fig. 3C). The spectrum of the UV-aged NPs also showed a peak in the 1200–950 cm^−1^ region associated with alkoxy groups and a peak in the 950–750 cm^−1^ region associated with C–H groups (Tasumi 2014). The results of our current study have been previously supported and indicate that photooxidation can produce oxygen-containing groups (Tasumi 2014; Wang et al. 2019; Doğan^ 2021^; Phan et al. 2022). Importantly, these changes imply oxidation and modification of the functional chemistry after exposure to UV radiation.

### Metal sorption to particles

**Fig. 4:**
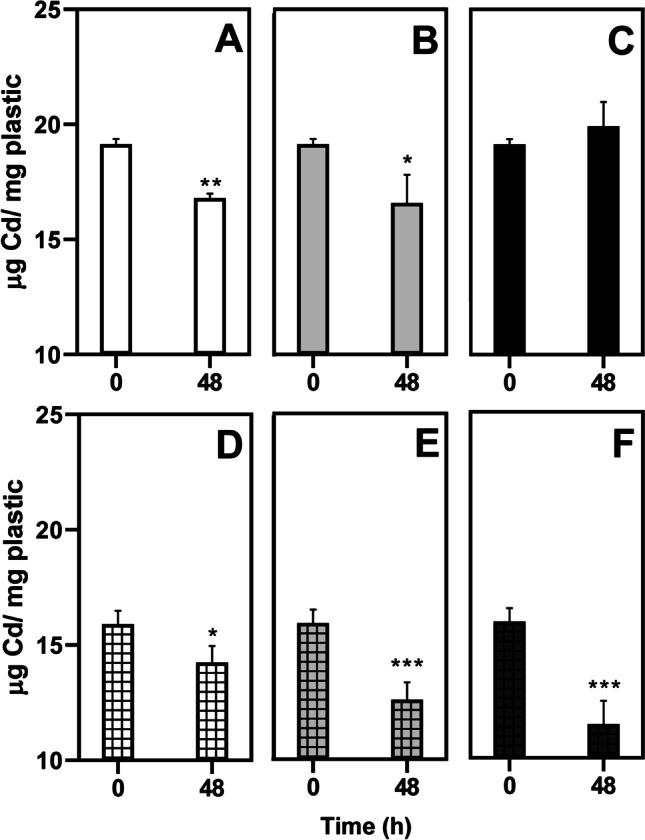
Dissolved Cd concentration (µg) per mg of NMPs following exposure for 48 h to 450 µg/L of Cd of 25 mg/L of non-UV-aged HDPE MPs (**A**), oxidized MPs (**B**), and NPs (**C**), and UV-aged MPs (**D**), oxidized MPs (**E**), and NPs (**F**). Data are presented as mean ± SD. Statistical differences from 0 h are indicated by asterisks (*, **, and *** correspond to *p* < 0.05, 0.01 and 0.001; Student’s t-test; *n* = 3)

In the present investigation, adsorption experiments were carried out with a concentration of Cd and NMPs of 450 µg/L and 25 mg/L, respectively. Figure 4 shows the sorption of Cd up to 48 h of the six different types of particles tested. Previous studies showed the adsorption of Cd to HDPE with different particle sizes (Wang et al. 2019). Consistently, in our study, the concentration of dissolved Cd decreased by 12.22 and 13.36% in the assays with plain (Fig. 4 A) and oxidized MPs (Fig. 4 B), respectively. The only experimental condition that showed no significant changes in the dissolved Cd concentration was that of NPs co-exposed with Cd at 48 h (Fig. 4 C). UV aging increased the sorption properties of NMPs, resulting in a decreased concentration of dissolved Cd of 10.48%, 20.86%, and 27.87% for MPs (Fig. 4 D), oxidized MPs (Fig. 4E), and NPs (Fig. 4F), respectively. Furthermore, desorption results showed values of 2.72 µg, 2.77 µg, and 1.53 µg of Cd/mg of non-UV-aged MPs, oxidized MPs, and NPs, respectively (Fig. S4). In the case of UV-aged particles, the results indicated values of 1.09 µg, 2.24 µg, and 2.92 µg of Cd/mg of plastic for MPs, oxidized MPs, and NPs, respectively (Fig. S4).

The sorption capacity of MPs for pollutants is highly influenced by pH, salinity, dissolved organic matter, hydrophobicity of pollutants, particle size, and physicochemical properties of the particles surface (Amelia et al. 2021; Bhagat et al. 2021). In previous studies, HDPE has been found to exhibit a relatively lower affinity for metals compared to other plastic materials (Holmes et al. 2012; Rochman et al. 2014). In this regard, the present investigation used the exposure medium L-15/ex, with Cd completely dissolved and predominantly present as CdCl^+^ and Cd^2+^, which could enhance the reaction with negatively charged plastics (Oldham et al. 2023). Furthermore, it has been noted that aged PE particles demonstrate a higher affinity for metals compared to non-aged PE (Holmes et al. 2012; Rochman et al. 2014). Other surface chemistry changes detected on the particles after photooxidation can affect the sorption of metals; for example, surface charge, increased porosity, which amplifies the surface area-to-volume ratio, and the oxygen-containing groups (Holmes et al. 2012; Brennecke et al. 2016; Wang et al. 2020a; Fu et al. 2021). Therefore, surface charge and particle size of both UV- and non-UV-aged plastic particles were examined to determine if there were any alterations in these parameters when exposed to Cd.

The addition of Cd significantly increased the agglomerated size of UV- and non-UV-aged oxidized MPs by approximately 4- and sevenfold (27.257 ± 17.783 µm UV-aged Ox MPs + Cd and 46.274 ± 12.590 µm non-UV-aged Ox MPs + Cd) and that of non-UV-aged NPs by 11-fold (8.383 ± 1.238 µm non-UV-aged NPs + Cd) (Fig. 1A). In this regard, a previous study indicated that NP size increased after Cd/Zn addition (Singh et al. 2019). Interestingly, co-exposure to 450 µg/L of Cd increased the surface charge on all particle types and treatments (Fig. 1B), this being significant with approximately a twofold increase in UV-aged MPs (− 9.35 mV) and non-UV-aged MPs (− 7.02 mV) and UV-aged oxidized MPs (− 13.00 mV), and with fourfold and 11-fold increases in non-UV-aged NPs (− 5.13 mV) and UV-aged NPs (− 2.00 mV), respectively. Similar results were obtained after co-exposure of several metals with PE, PS, and polytetrafluoroethylene NMPs, producing a significant rise on the particles charge (Dong et al. 2019; Zhou et al. 2021). The low zeta potential displayed by NMPs could accelerate the adsorption of cationic ions, such as Cd^+2^, due to the electrostatic attraction, neutralizing its surface charge and explaining the higher zeta potential values obtained after the metal addition (Tourinho et al. 2019; Gao et al.^ 2021^; Sun et al. 2021; Zhou et al. 2021). This can be translated into a different behavior of the particles in the media, producing a rapid homoaggregation of the NMPs and a potential prevention of the particles from interacting with the cells (Sun et al. 2021).

To further prove that Cd was adsorbed to the particles, SEM and EDS analyses were conducted. The results revealed from negligible to 0.90% of Cd sorbed onto the NMPs (Figs. S5–10). The desorption experiment results suggest that undetectable Cd on NMPs in certain cases via EDS might be due to low Cd sorption, falling within or below the equipment’s 0.1% detection limit (Reed 1997). Nonetheless, the ATR-FTIR analyses demonstrated the adsorption of Cd to NMPs, as it is possible to observe in the changes obtained in the spectra of the particles after making it react with Cd (Fig. 3). All particles presented modifications in the 1500–1300 cm^−1^ and 1300–850 cm^−1^ regions, showing widened peaks, which indicates less symmetric C–H bending, and a decrease in the signal of alkoxy groups appearing in the UV-aged particles with Cd (Tasumi 2014). In the case of oxidized MPs’ and NPs’ functional chemistry (Fig. 3B and C), the reaction with Cd also altered the 850–700 cm^−1^ region, producing a decreasing C–H bend signal. Also, modifications in the NPs 1900–1500 cm^−1^ region were observed, presenting widened C = O signal in the UV-aged NPs that reacted with Cd (Fig. 3C), indicating a potential reaction site (Tasumi 2014). Overall, more pronounced changes in the UV-aged oxidized MPs were observed. These results agree with the changes in the spectra after Cd addition observed previously in the literature (Wang et al.^ 2019^; Guo et al. 2020). The zeta potential results obtained added to the observation of the ATR-FTIR, which suggests no new peak formation due to the presence of Cd in the medium, might indicate that the dominant sorption mechanism is physical (Chen et al. 2018; Guan et al. 2018; Guo et al. 2020). Previous studies with Cd and HDPE NMPs have shown that Van der Waals forces dominate this sorption mechanism, potentially resulting in slower or faster desorption of contaminants which can further affect bioaccumulation and toxicity (Wang et al. 2019; Gao et al. 2021).

### Quantification of Cd bioaccumulation

**Fig. 5:**
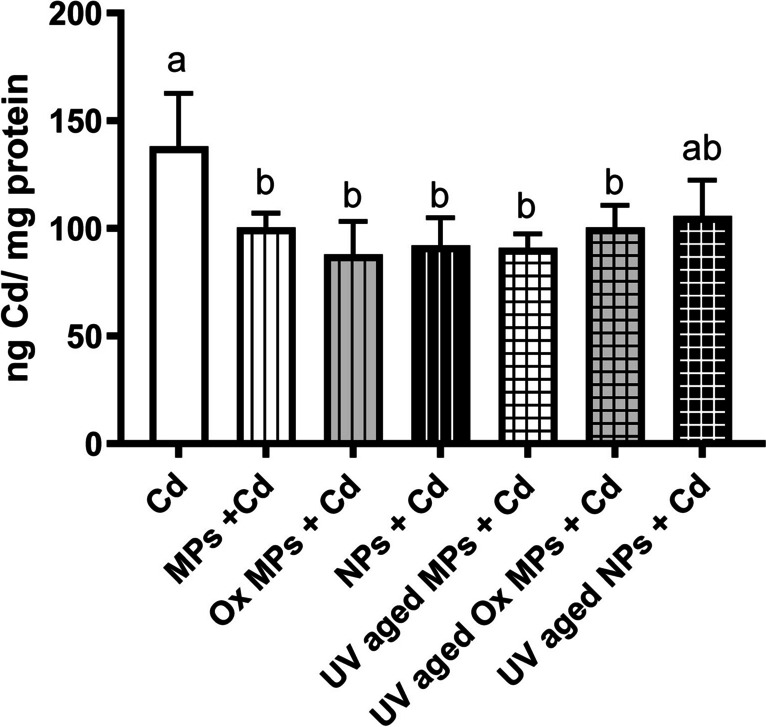
Bioaccumulation of Cd (ng) normalized by mg of protein content in RTgutGC cells after exposure for 24 h to 450 µg/L of Cd alone and combined with 25 mg/L of UV and non-UV-aged microplastics and nanoplastics (NMPs). Data are presented as mean ± SD. Bars bearing different letters are significantly different (one-way ANOVA, Tukey’s multiple comparison test; alpha = 0.05; *n* = 4)

Figure 5 shows Cd accumulation in RTgutGC cells after 24-h exposure to 450 µg/L of Cd alone and in co-exposure with the UV-aged and non-UV-aged particles. Cadmium concentrations reached values of 138.14 ± 24.71 ng Cd/ mg protein in RTgutGC cells exposed in absence of particles and ranged from 87.91 to 105.73 ng Cd/ mg protein in cells co-exposed with NMPs. Therefore, in cells exposed to all NMP types, a reduction is established, although not significant for UV-aged NPs, in Cd accumulation. Notably, UV-aged oxidized MPs and NPs sorb more Cd than the respective not UV-aged particles (Fig. 4). No differences in bioaccumulation were observed in oxidized MPs, but the slightly higher Cd accumulation in cells co-exposed to Cd and UV-aged NPs could be explained by the fact that internalization of particles in the size range under 500 nm can enter cells via endocytosis whereas bigger particles cannot (Rejman et al. 2004).

Bioaccumulation of pollutants sorbed to NMPs in an organism depends on several factors, such as the physicochemical properties of the particles, desorption rate of the pollutant from the particle, organism internal environment, and retention time (Barboza et al. 2020; Ma et al. 2020; Amelia et al. 2021). In this regard, the literature describes five possible bioaccumulation scenarios of pollutants introduced by these particles into organisms (Tourinho et al. 2019; Amelia et al. 2021). The results of the present study show that co-exposures with NMPs reduces Cd bioaccumulation in RTgutGC cells indicating that HDPE NMPs can sorb Cd, thus decreasing its bioavailability in solution and its bioaccumulation in RTgutGC cells (Tourinho et al. 2019; Amelia et al. 2021). This effect has been previously attributed to pre-incubation of the pollutant with the particles before exposure, which may produce a strong sorption to the plastic and low desorption during the exposure period (Tourinho et al. 2019).

In this investigation, pre-sorbed particles for 48 h before cell incubation led to reduced Cd bioaccumulation when NMPs were present. This phenomenon was also observed in zebrafish (*Danio rerio*) and blue discus (*Symphysodon aequifasciatus*) exposed to PE MPs + silver (Ag) and PS MPs + Cd, respectively, indicating that pre-incubation of metals with plastic prior to exposure resulted in lower Ag and Cd bioaccumulation (Khan et al. 2015; Wen et al.^ 2018^). Nonetheless, studies in which Cd was not pre-sorbed with the plastics have found higher accumulations of Cd in grass carp (*Ctenopharyngodon idella*) and zebrafish (Yang et al.^ 2022^; Zuo et al. 2022). Thus, pre-incubation appears to be a key factor affecting metal bioaccumulation on fish. Moreover, the type and size of NMPs may change the resulting interactions with metal pollutants and the cells. Furthermore, the type of plastic used in this study (HDPE) has a physical characteristic highly relevant to the bioaccumulation potential: its density. Although sonicated and well mixed prior to exposure, particles’ low density causes floating, limiting cell interaction and potentially impeding Cd bioaccumulation.

Several studies have highlighted a higher accumulation of contaminants produced by smaller particles due to its higher area-to-volume ratio (Tallec et al. 2018; Tourinho et al. 2019; Kögel et al. 2020; Sun et al. 2022). In this regard, DLS and zeta potential measurements also indicated that NPs aggregated and produced agglomerates that can lead to higher sorption of toxicants (Pittura et al.^ 2018^; Zhang et al. 2018; Trevisan et al. 2019; Zhu et al. 2019; Bhagat et al. 2021). However, at the same time, the large size of the MPs employed and the NMP aggregates formed can be expected as a limitation for internalization through endocytosis and, therefore, for potential Cd bioaccumulation in the cells (Augustine et al. 2020; Bhagat et al. 2021; de Almeida et al. 2021). Therefore, size would explain, to some extent, the lower bioaccumulation of Cd found in the cells when plastics were present in the media and the slight differences in bioaccumulation among different particles’ sizes.

Even though in this study HDPE NMPs appear to sorb Cd, it has been highlighted in the literature the capacity of this metal to be released from the particles when the environmental factors change. Thus, the loaded particles’ presence in the acidic pH of the organism internal environment (e.g., in the stomach) may cause the desorption of Cd from the plastic, becoming a potential threat for the individual (Wang et al. 2019; Maity et al. 2021). Also, considering the density of these particles, it is noteworthy that the bioaccumulation potential can differ for pelagic species that feed in surface waters and the neustonic layer of aquatic systems (Collignon et al. 2012).

### Cytotoxicity assays

In the current investigation, RTgutGC cells were exposed to NMPs at concentrations ranging from 12.5 to 200 mg/L for 24 h. Although the majority of the concentrations tested in this study exceeded the levels commonly reported in the environment (Collignon et al. 2012; Kang et al. 2015; Reisser et al. 2015; Green et al. 2017), it is crucial to acknowledge the lack of accurate environmental concentrations due to insufficiency of existing data regarding particles’ concentrations in aquatic systems below 300 μm and about the weathering status of particles, Additionally, predictions anticipate a rise in concentration levels in the future, emphasizing the importance of investigating higher concentrations in toxicological studies (Koelmans et al. 2016; Almeida et al. 2019; Oliveira et al. 2019; Garcia-Muñoz et al. 2023).

The spectrogram of FTIR verified that NMP composition was high-purity polyethylene. Therefore, all cytotoxicity effects can be attributed solely to pure HDPE and no other sources of contamination can be expected. Statistical analyses for metabolic activity, plasma membrane integrity, and lysosomal integrity did not indicate any significant differences among treatments (i.e., 12.5–200 mg/L of NMPs) and the control for any of the concentrations and particles tested (Fig. S11). Therefore, it can be assumed that HDPE MPs and NPs are not affecting RTgutGC cells in terms of metabolic activity, plasma membrane, and lysosomal integrity at the concentrations tested.

Microplastics and NPs ingested by aquatic animals can accumulate in their mandible, stomach, gut, liver, kidney, appendages, gills, and muscles, among others, potentially causing alterations in gene expression, gastrointestinal function and physiology, and immune responses. Additionally, they can induce oxidative stress, cytotoxicity, neurotoxicity, and disruptions in reproduction, growth, behavior, and survival (Zaki and Aris 2022; Osman et al. 2023). Research on fish exposed to PE MPs, both aged and non-aged, is scarce, especially at the cellular level. Therefore, comparisons with our results at the cellular level are difficult. However, previous studies using six different mammalian cells exposed to PE MPs (2 or 30 μm) have shown effects on cell viability in intestinal (Caco-2) and lung (A549) cells, but only at very high concentrations, such as 1000 mg/L (Gautam et al. 2022). Another study using Madin–Darby canine kidney (MDCK) cells has shown inhibition of cell viability by PE MPs (1–4 μm) at lower concentrations (1 mg/L) (Palaniappan et al. 2022). After exposing human choriocarcinoma (BeWo b30) cells to 0.1–100 mg/L of aged and non-aged HDPE MPs (0–80 μm), no effects on viability and plasma membrane integrity were detected (Dusza et al. 2022). Thus, although the acute toxicity of PE appears to be generally low, similarly to our study, particle size and cell type affect the toxic response. Studies on PS toxicity are more abundant and have been extended to include investigations in fish cell lines as well. For instance, PS NPs (100 nm) showed low acute effects on cell viability, but revealed an inhibitory effect on antioxidant enzymes such as glutathione-S transferase and catalase in fibroblast cells derived from seabream (*Sparus aurata*) and neuronal cells derived from sea bass (*Dicentrarchus labrax*) (Almeida et al. 2019). Moreover, functionalized PS NPs of 50 nm (pristine, amino, and carboxylic) showed that only the amino-functionalized PS NPs affect cell viability of a brain-derived cell line (SaB-1) from seabream (González-Fernández et al. 2021). PS MPs (2 µm sized) increased the cytotoxic potential in a murine liver macrophage (ImKC), including effects on metabolic activity, membrane integrity, genotoxicity, and ROS formation, particularly after exposure to 130-day aged MPs compared to non-aged MPs (Völkl et al.^ 2022^). Furthermore, PET and polyvinyl chloride (PVC) MPs (25 and 90 μm) did not induce any inhibition of cell viability in three rainbow trout cell lines derived from the gill (RTgill-W1), liver (RTL-W1), and gonads (RTG-2), but PVC induced the formation of reactive oxygen species (ROS) (Boháčková et al. 2023). These studies suggest that the impact of different plastic types on cell viability is low, but effects at the enzymatic level can occur and are related to the plastic type.

In the present study, a substantial proportion of the total tested particles displayed a relatively large size. This, in conjunction with the formation of aggregates, results in an effective prevention of the internalization and potential toxicity of NMPs (Rejman et al. 2004; Gustafson et al. 2015; Augustine et al. 2020; Bhagat et al. 2021; de Almeida et al. 2021). After internalization, particles can affect lysosomal stability and generate ROS, leading to mitochondrial damage (Gustafson et al. 2015). However, our data indicate that there was no effect on cell membrane integrity or lysosomal integrity, suggesting that these HDPE MPs are likely not entering the cells, with only a minor fraction of NPs having the necessary size for entering cells.

**Fig. 6:**
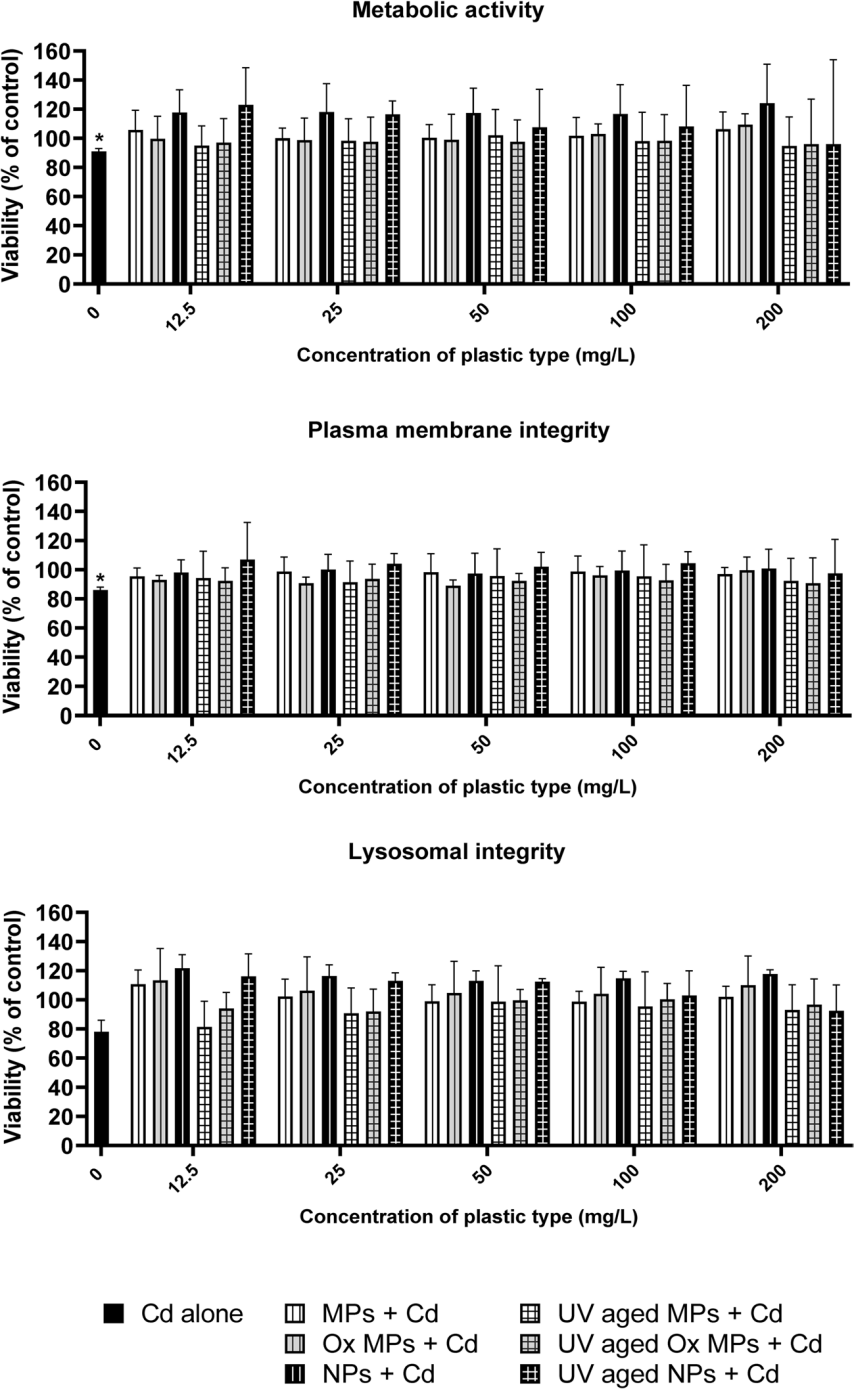
Effects on metabolic activity, plasma membrane, and lysosomal integrity after 24 h exposure to a range of concentrations of UV and non-UV weathered HDPE NMPs (12.5–200 mg/L) co-exposed with 450 µg/L of Cd on RTgutGC cells. Data are presented as mean ± SD, reported as viability percent of control. Statistical difference between all NMPs co-exposures compared to Cd alone (450 µg/L of Cd) was tested but no difference was found (ANOVA, Dunnett’s multiple comparison test; alpha = 0.05; *n* = 3–4). Unpaired *t*-test with Welch correction were performed on fluorescence values to test statistical differences between negative control (L-15/ex) and all treatments, and statistical differences are indicated with * (alpha = 0.05; *n* = 3–4)

The presence of NMPs can not only influence the bioaccumulation of Cd in RTgutGC cells but ultimately may also modulate the toxic effects of Cd. Moreover, as previously described, the sorption of pollutants to NMPs may become a threat to biota after ingestion, which warrants further investigation (Tourinho et al. 2019). While exposure to 450 µg/L of Cd alone showed significant reduction of metabolic activity and plasma membrane integrity, the co-exposure experiments of the different plastic particles with 450 µg/L of Cd resulted in no significant acute toxic effects in any of the three endpoints tested (Fig. 6). Hence, it can be concluded that these HDPE NMPs do not influence Cd toxicity in RTgutGC cells, at least not for cell metabolic activity, membrane integrity, and lysosome integrity at the examined concentrations. Nevertheless, the possibility of effects on other endpoints, such as apoptosis, gene expression, oxidative stress, and other biomarkers, cannot be dismissed (Lu et al. 2018; Wen et al. 2018; Chen et al. 2022). Further studies are required to explore more in depth the mechanism of toxicity of NMPs, both alone and in combination with Cd. Besides the size, the inherent low density of the particles, and the incipient aggregation displayed by all particles after Cd addition can produce a potential prevention of the NMPs from interacting with the cells. Furthermore, the sorption of Cd to the NMPs surface may result in a reduction of the bioavailable Cd in solution, which is consistent with the drop in toxicity reported in this study. Accordingly, a previous study in common goby (*Pomatoschistus microps*) have found also lower Cd toxicity when 1.5 to 0.012 mg/L of 1–5 µm PE NMPs were present (Miranda et al. 2019). Another study observed how common carp (*C. carpio*) experienced increased toxicity when Cd was exposed with 0.25–0.5 mg/L of PE MPs of unspecified particle sizes (Banaee et al. 2019). The outcome of the present study may differ with particles of varying density, highlighting the need for further studies involving different types of plastics.

Although this study primarily focused on acute exposures, it is crucial to consider the potential long-term environmental effects and ecological relevance. While acute adverse effects may not have been observed, the interactions between NMPs alone or in combination with contaminants could have more significant consequences over time. For instance, in mouse embryonic fibroblasts, 6 months chronic exposure to 10–100 μg/mL of 50 nm PS NPs resulted in tumoral phenotypes appearing in exposed cells (Barguilla et al. 2022). Also, previous chronic studies in zebrafish exposed to 20 μg/mL of PE MP (13.5 μm) and NPs (70 nm), and to 20–200 µg/L 5 μm PS MPs + 10 μg/L Cd for up to 3 weeks have shown that presence of plastic particles lead to neurotoxicity, oxidative damage, and inflammation, and enhanced the toxicity of Cd (Lu et al. 2018; Li et al.^ 2023^). These changes could potentially impact feeding, behavior, growth, reproduction, and survival rates in fish (Salerno et al. 2021), leading to cascading effects on aquatic ecosystems, which might disrupt food webs and threaten the overall ecosystem stability.

## Conclusions

Our investigation provides new insights about toxicity and potential bioaccumulation of contaminants in fish intestinal cells due to HDPE NMPs with varying physicochemical properties. Particle characterization in this study showed overall incipient stability, except for Cd-coated particles, which rapidly aggregated. ATR-FTIR analyses indicated oxidation and modification of the functional chemistry after exposure to 6 weeks of UV radiation. SEM analysis of the UV-aged plastic particles reported notable alterations on their surface, presenting more roughness and a damaged morphology. Overall, higher differences of Cd sorbed to UV-aged particles were detected after 48 h, consistent with the increased surface charge, increased roughness, and the oxygen-containing groups detected on the particles after weathering. NMPs in the exposure medium decreased Cd bioaccumulation in RTgutGC cells, except for UV-aged NPs, which exhibited comparable Cd bioaccumulation to that of waterborne Cd. Data indicated no toxicity on RTgutGC cells exposed to UV- and non-UV-aged NMPs alone. Moreover, in Cd NMP co-exposures, NMPs attenuated the toxicity of Cd. Although in the present study, the co-exposure of plastics with Cd reduced the bioavailability of the metal, chronic exposure to plastics particles produced Cd toxicity enhancement in other fish species. Therefore, the need for stricter regulations on NMP production and release in the environment, the requirement for chronic exposure assessments in environmental risk evaluations, and mitigation strategies to ensure the protection of aquatic ecosystems is of vital importance.

### Supplementary Information

Below is the link to the electronic supplementary material.

Supplementary file1 (DOCX 1832 KB)

## Data Availability

Authors can confirm that all relevant data are included in the article or in the online resources provided. The raw datasets used and/or analyzed during the current study are available from the corresponding author on reasonable request.
