# Supplementary material for: Role of UV radiation and oxidation on polyethylene micro- and nanoplastics: impacts on cadmium sorption, bioaccumulation, and toxicity in fish intestinal cells
Source: Environ Sci Pollut Res Int. 2024 Jul 17;31(35):47974–90. doi: 10.1007/s11356-024-34301-x (PMC11297841; doi:10.1007/s11356-024-34301-x)
Supplement: Supplementary file 1 — Supplementary file1 (DOCX 1832 KB) [file 11356_2024_34301_MOESM1_ESM.docx]

**SUPPORTING INFORMATION**

Role of UV Radiation and Oxidation on Polyethylene Micro- and Nanoplastics: Impacts on Cadmium Sorption, Bioaccumulation, and Toxicity in Fish Intestinal Cells

Estefanía Pereira Pinto, Justin Scott, Kendra Hess, Estefanía Paredes, Juan Bellas, Jorge Gonzalez-Estrella & Matteo Minghetti

Corresponding author

E-mail address: [estefania.pereira.pinto@uvigo.es](mailto:estefania.pereira.pinto@uvigo.es) (E. P. Pinto)

**This Supporting Information contains:**

- **Page S2:** Cadmium concentration in solution after 48 h incubation with MPs/NPs, **Fig. S1**
- **Page S3:** Cadmium concentration in solution at 0, 6, 24 and 48 h incubation with MPs/NPs**, Fig. S2**
- **Page S4:** Oxidized MPs in Ultrapure water (UPW) and in L-15/ex**, Fig. S3**
- **Page S5:** Desorption results of Cd from MPs/NPs, **Fig. S4**
- **Page S6-11:** EDS analysis of the HDPE MPs/NPs used in this study, **Fig. S5-S10**
- **Page S12:** Effects on metabolic activity, plasma membrane and lysosomal integrity of UV and non-UV weathered HDPE MPs/NPs, **Fig. S11**
- **Page S13:** Carbonyl index calculated for NMPs before and after UV aging. **Table S1**
- **Page S14-15:** Details regarding sample preparation, measurement, and reagents information of MPs/NPs characterization. **Text S1**


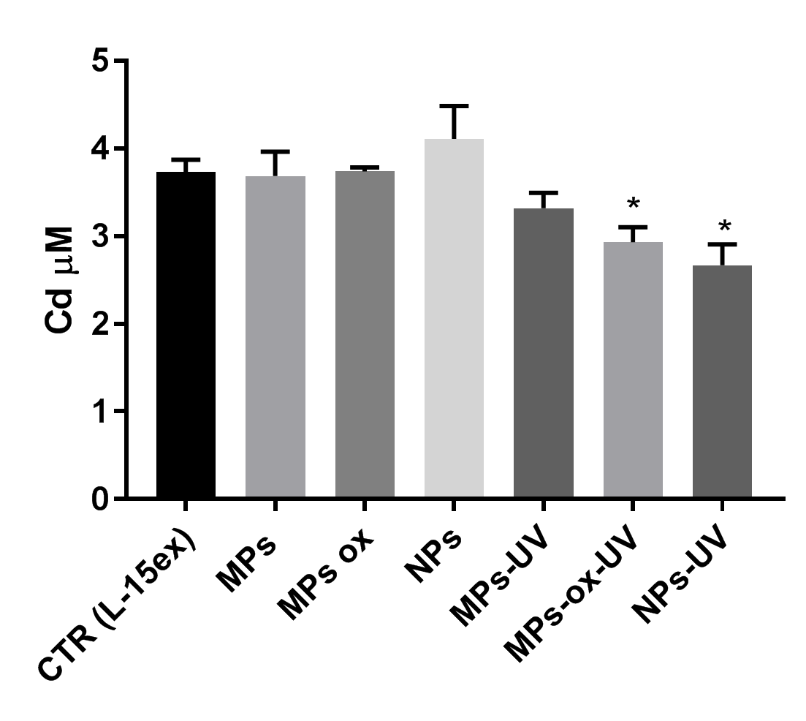


**Fig. S1** Cadmium concentration in solution after 48 hours incubation with MPs/NPs. In the control, cadmium was dissolved in MPs/NPs free L-15/ex. Statistical difference from control is indicated by an asterisk (one-way ANOVA, Dunnett’s multiple comparison test; alpha = 0.05; n = 3)

**
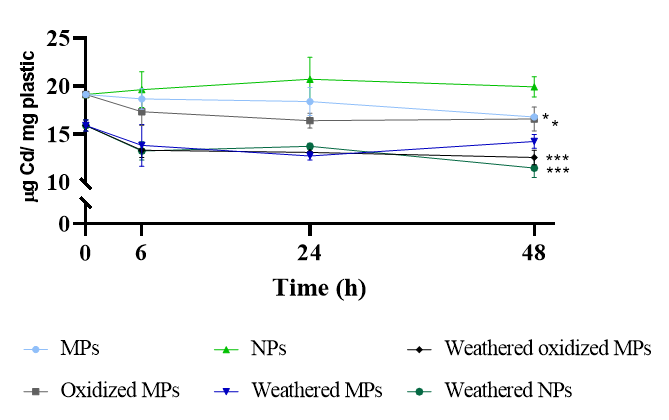
**

**Fig. S2** Cadmium concentration in solution at 0-, 6-, 24- and 48-hours incubation with MPs/NPs. At 0 h, cadmium was dissolved in MPs/NPs free L-15/ex. Statistical difference from 0 h is indicated by an asterisk (one-way ANOVA, Dunnett’s multiple comparison test; alpha = 0.05; n = 3)

**Fig. S3** Size (nm) of Oxidized MPs in Ultra-Pure Water (UPW) in comparison to Oxidized MPs in L-15/ex **(**n = 5)

**Fig. S4** Desorption results of Cd from microplastics and nanoplastics


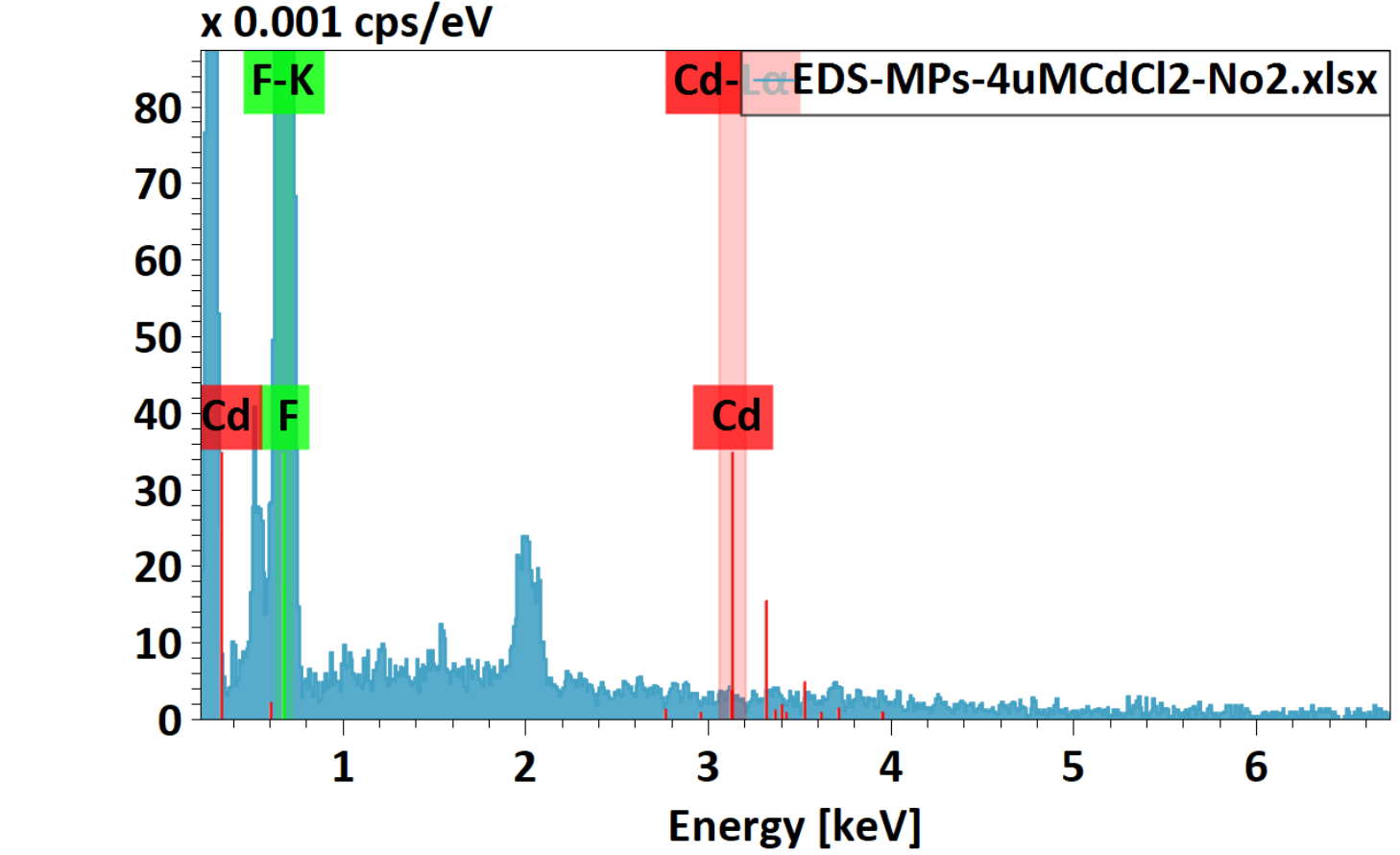

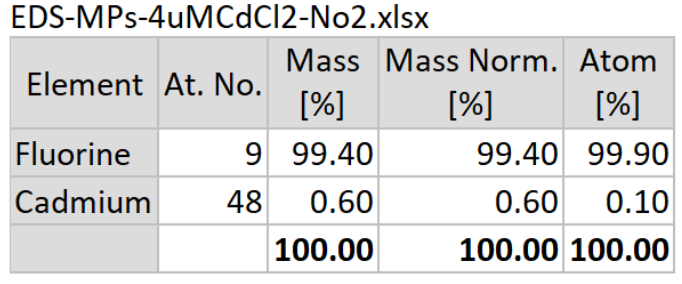


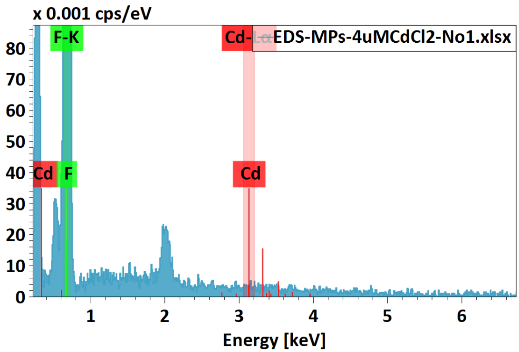

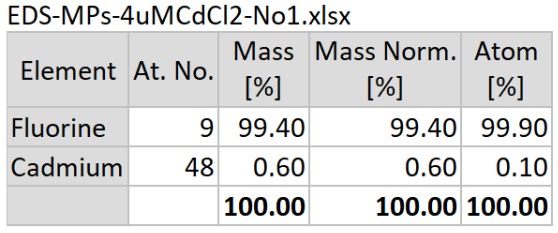


**Fig. S5** EDS spectrum of MPs alone and in combination with Cd showing low presence of Cd on the MPs surface


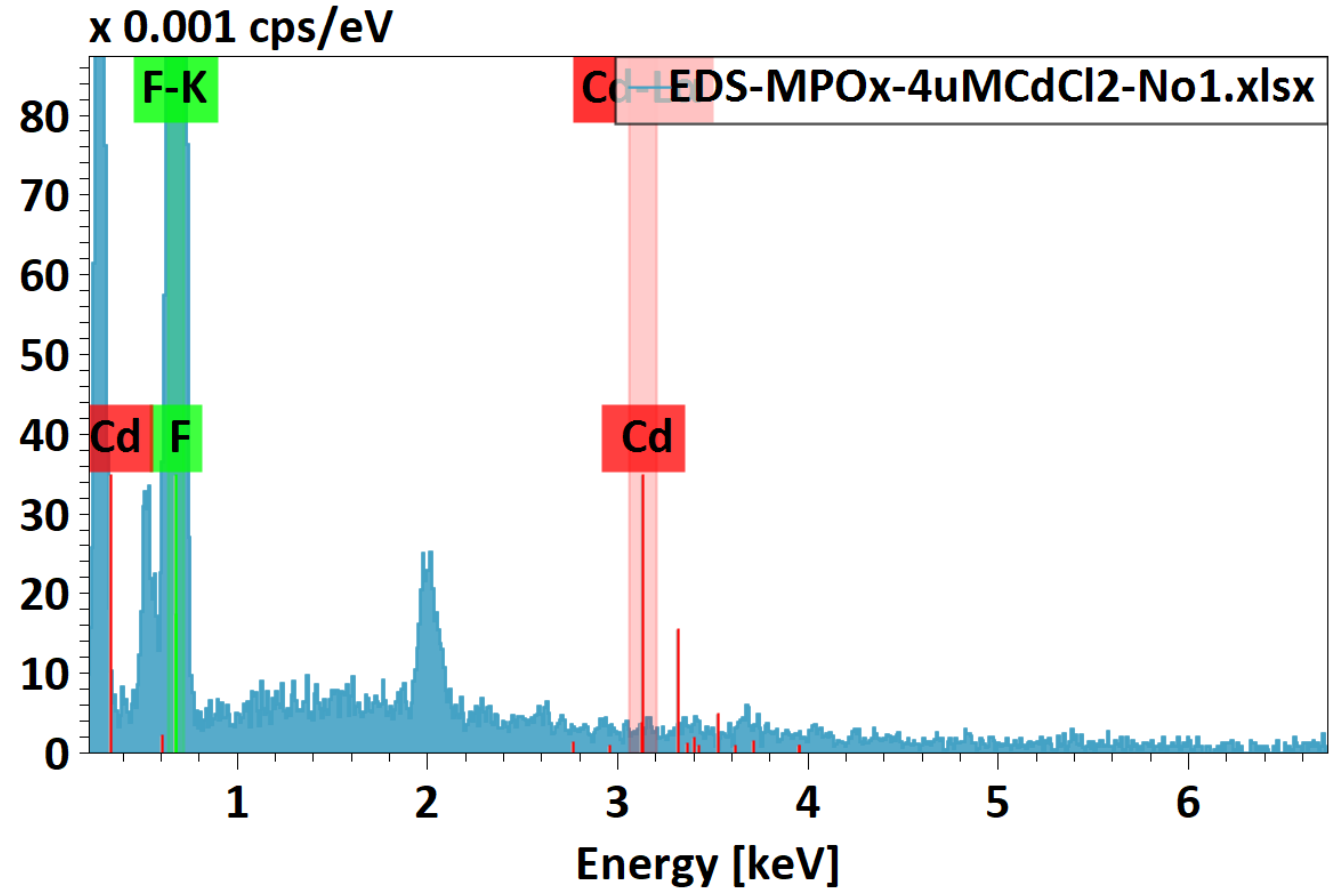

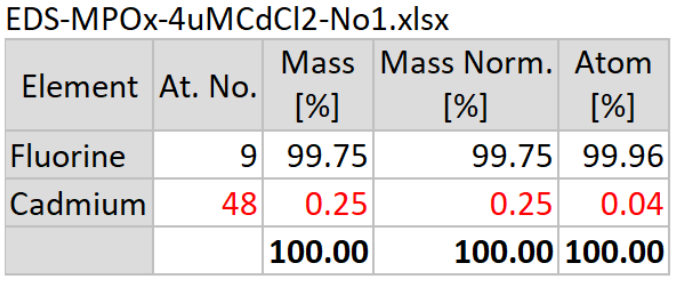


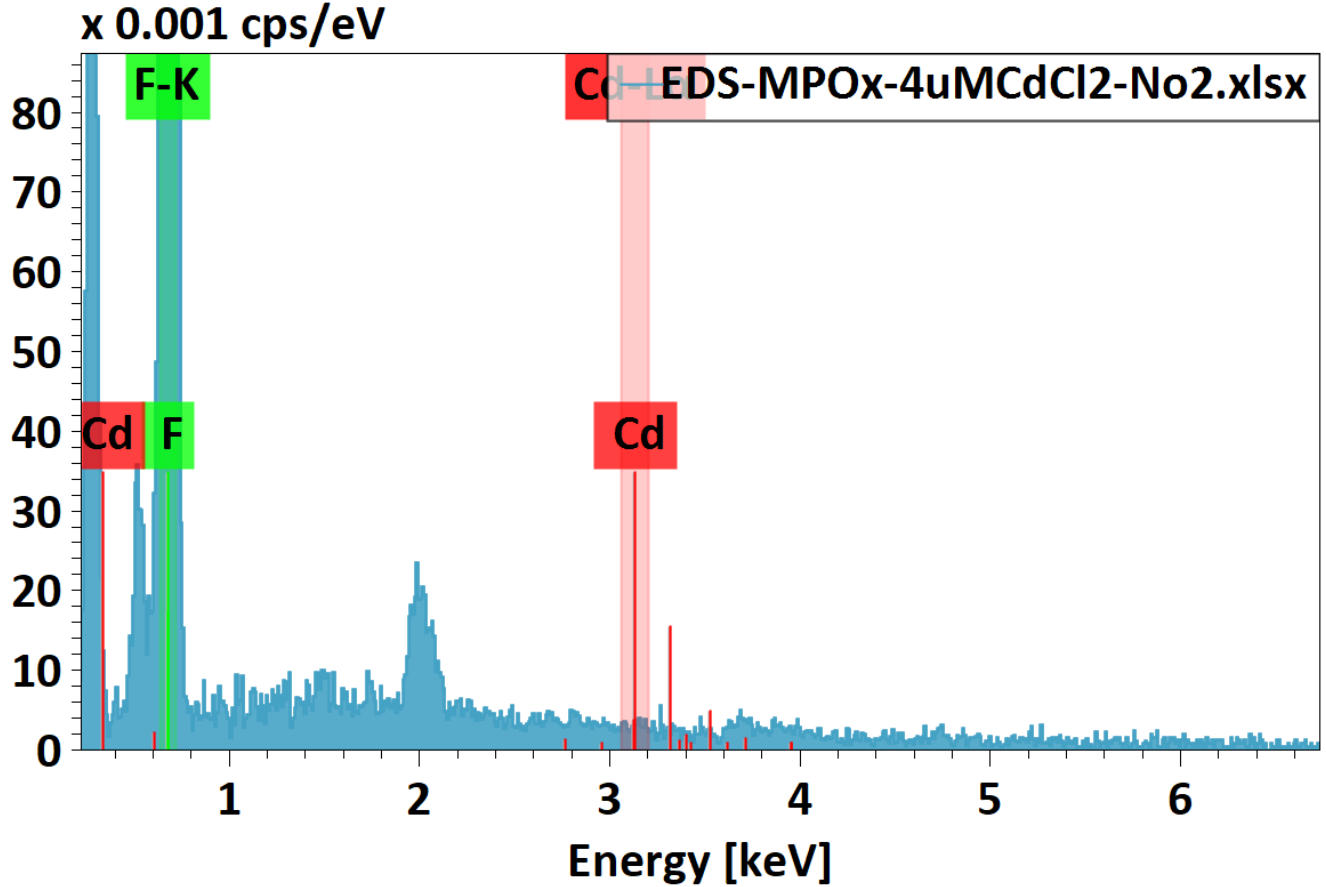

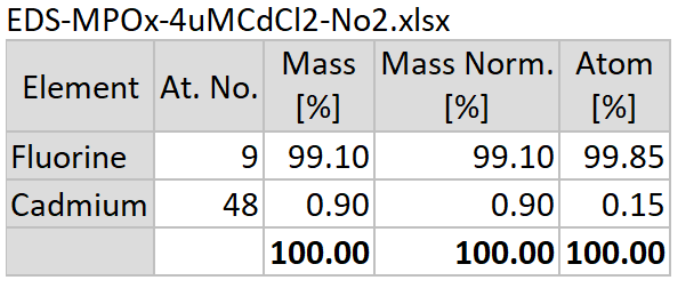


**Fig. S6** EDS spectrum of oxidized MPs alone and in combination with Cd showing low presence of Cd on the oxidized MPs surface


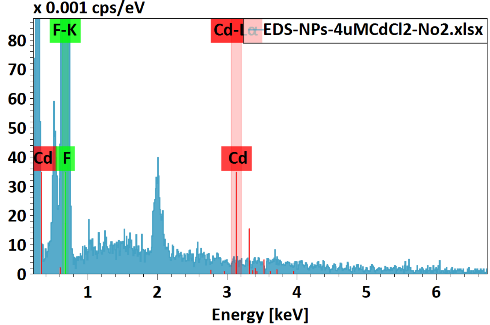

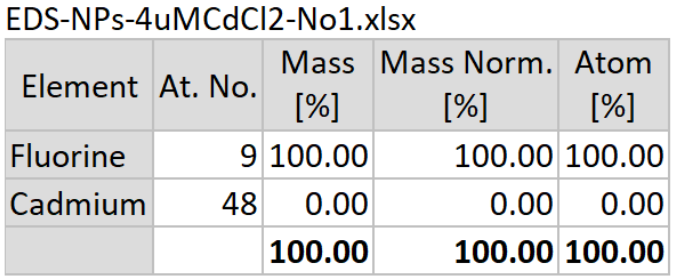

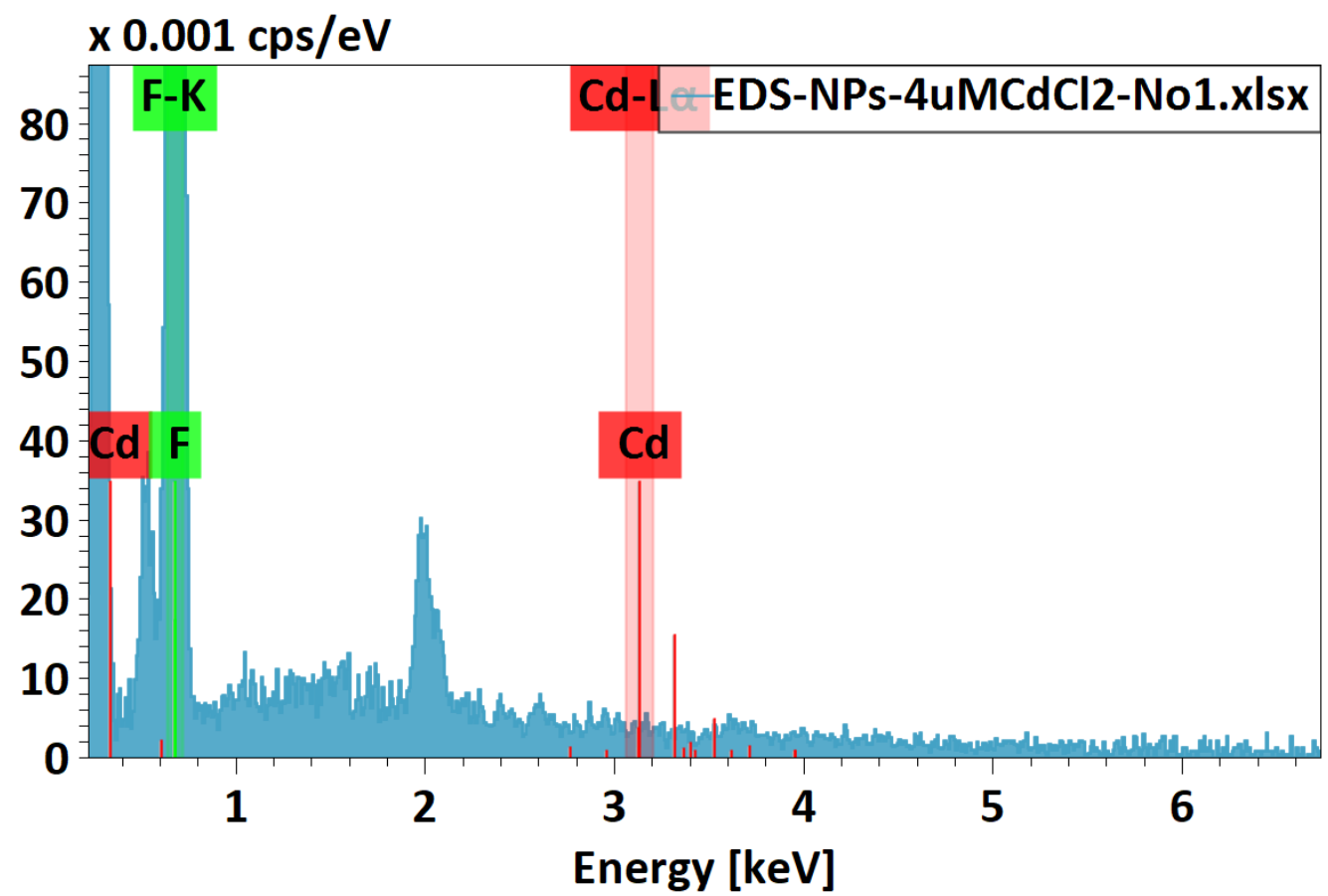


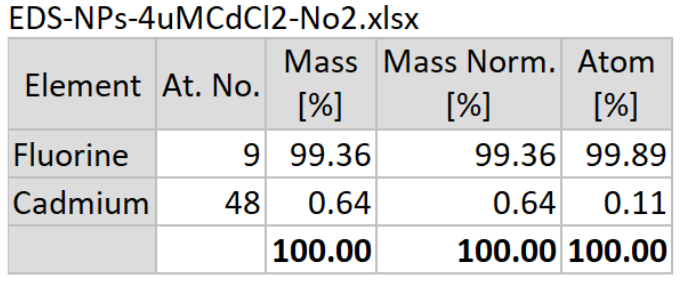


**Fig. S7** EDS spectrum of NPs alone and in combination with Cd showing low presence of Cd on the NPs surface


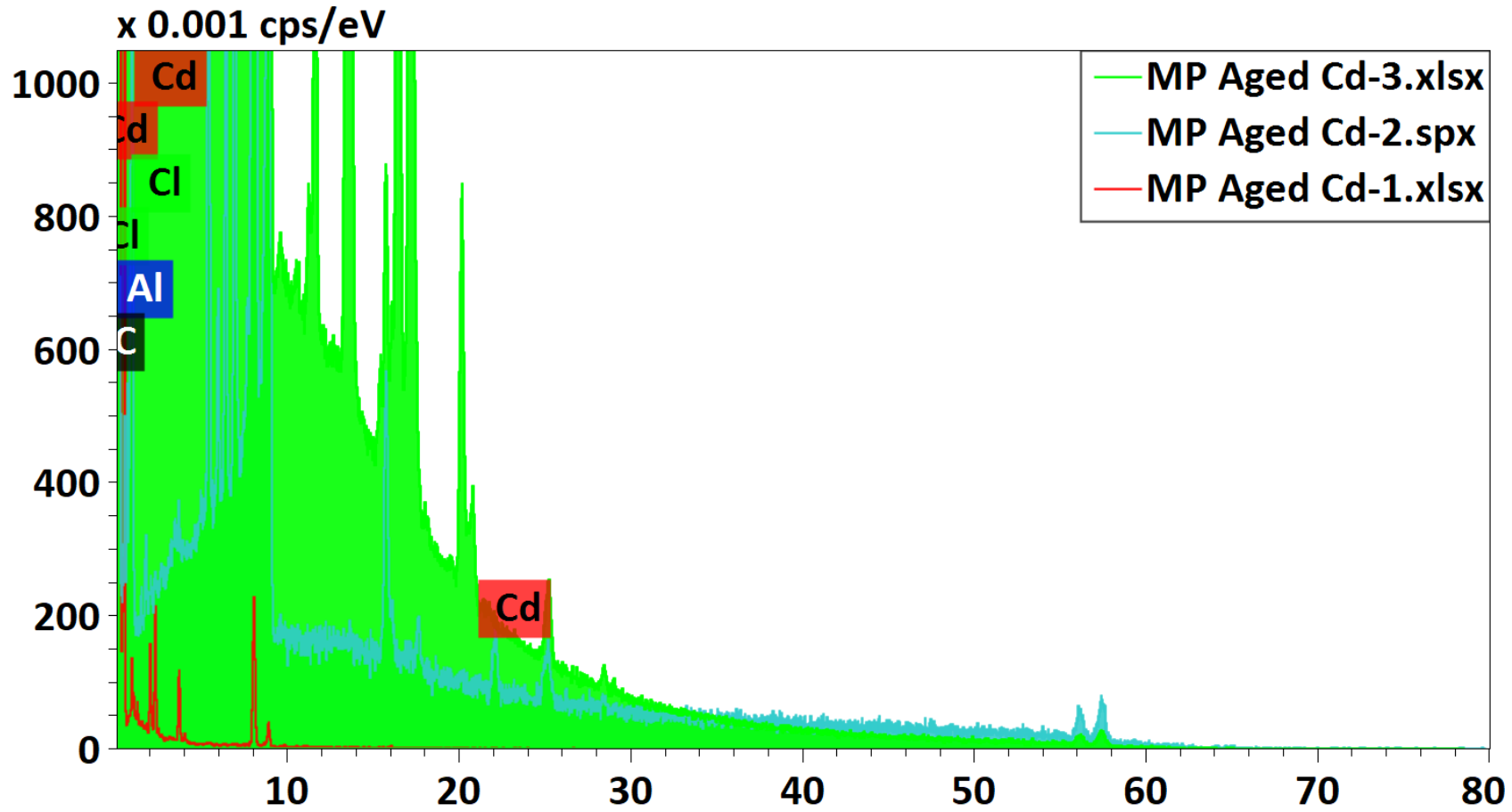

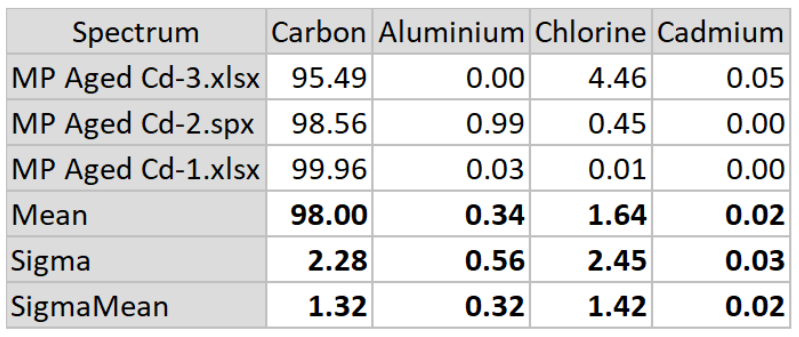


**Fig. S8** EDS spectrum of UV aged MPs alone and in combination with Cd showing low presence of Cd on the MPs surface


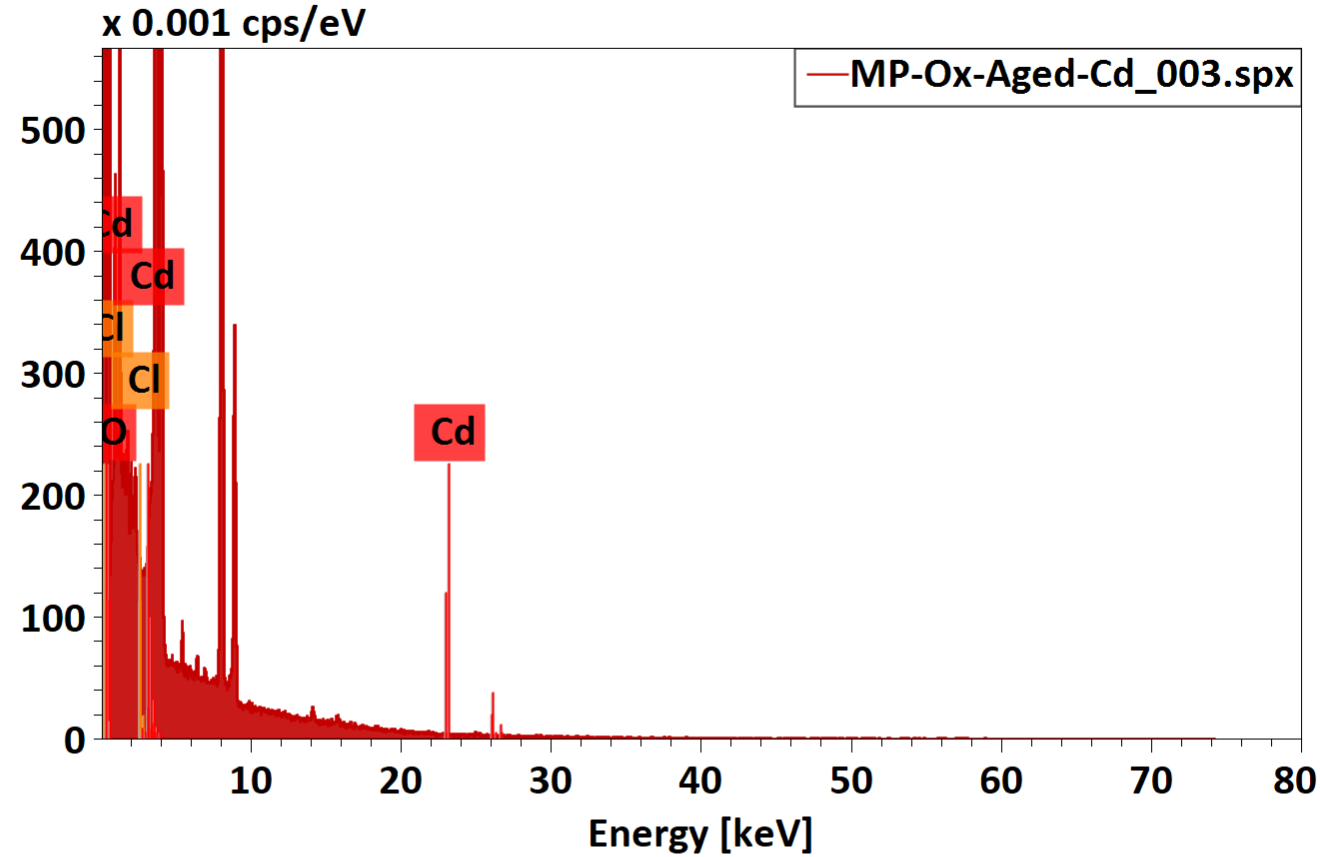


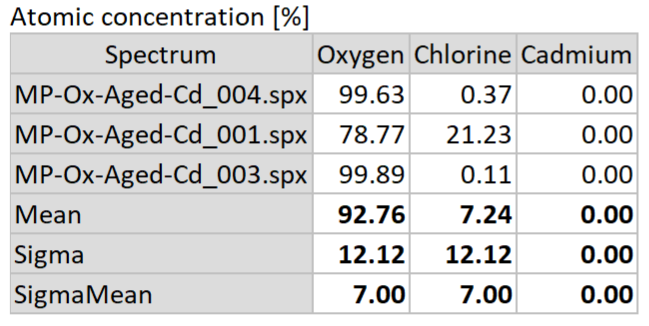


**Fig. S9** EDS spectrum of UV aged oxidized MPs alone and in combination with Cd showing no presence of Cd on the oxidized MPs surface


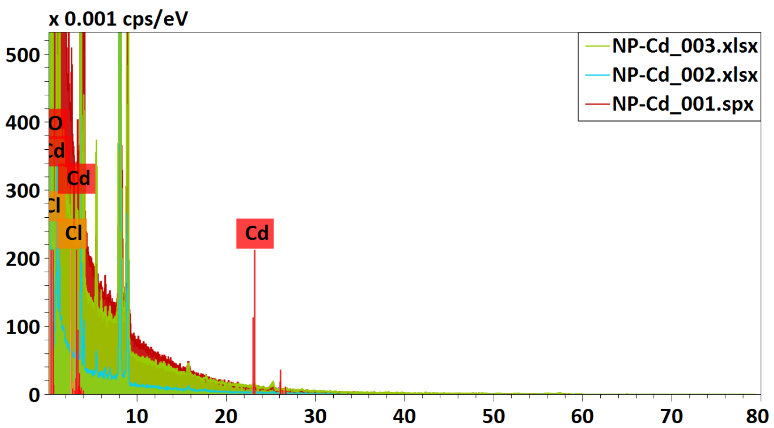


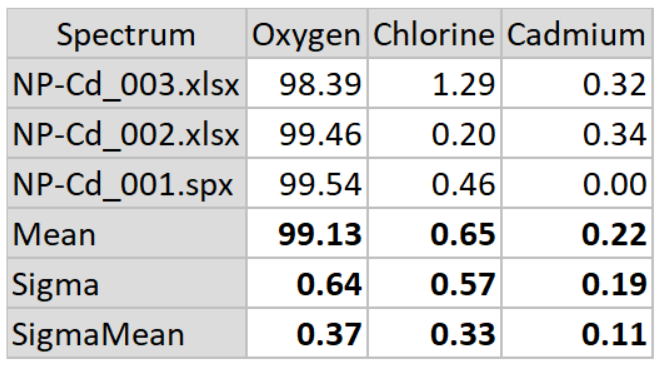


**Fig. S10** EDS spectrum of UV aged NPs alone and in combination with Cd showing low presence of Cd on the NPs surface

**Fig. S11** Effects on metabolic activity, plasma membrane and lysosomal integrity after 24 hours exposure to 2,700 µg/L of Cd (positive control) and to a range of concentrations of UV and non-UV weathered HDPE MPs/NPs (12.5-200 mg/L) on RTgutGC cell lines. Data are presented as mean ± SD reported as viability percent of control. Effects of plastic concentration and plastic type were tested separately but no statistical differences were detected (one-way ANOVA, Tukey’s multiple comparison test; alpha = 0.05; n = 3-4)

**Table S1** Carbonyl indices calculated for NMPs before and after UV aging

| **Material** | **CI** |
| --- | --- |
| Non-aged MPs | 0.0 |
| UV aged MPs | 0.12 |
| Oxidized MPs | 0.024 |
| UV + Oxidized MPs | 0.025 |
| Non-aged NPs | 0.016 |
| UV aged NPs | 0.027 |

**Text S1** Details regarding sample preparation, measurement, and reagents information of MPs/NPs characterization:

**Section 2.1. Plastic Particles and Weathering Process** A micronized powder (MPP 635-XF) was selected as the plain MPs and constitutes a mixture of different shapes and sizes (nominal size 2-10 µm) of polyethylene particles, with a melting point of 123-125 °C and a density of 0.97 g/cc (at 25 °C). The oxidized MPs selected was a micronized powder (Aquatex 325), which has a high molecular weight, exhibits nominal sizes in the 10–15 μm range and has a melting point of 135–140 °C and a density of 0.99 g/cc (at 25 °C). NPs provided by Cospheric were polyethylene nanospheres (PENS-0.95). The nominal size range of NPs has been established at 200-9,900 nm and density at 0.95-0.98 g/cc.

**Section 2.3.2. Scanning electron microscopy/energy dispersive X-ray spectroscopy.** Solutions of MPs/NPs, UV and non-UV aged, and with and without 450 µg/L Cd, were prepared at a concentration of 25 mg/L, as described above (section 2.2 of the main document). Solutions were applied directly to double-sided carbon tape on aluminum SEM supports and coated in gold/palladium or carbon to manage charging and reduce artifacts.

**Section 2.3.3.** **Attenuated Total Reflectance -** **Fourier Transformed Infrared Spectroscopy**. Identical samples to those prepared for SEM and zeta potential measurements were processed. To obtain the spectra, a Nicolet iN10-MX micro-FTIR (Thermofisher, Waltham, MA, US) was used in ATR mode with a cooled detector and germanium ATR tip. Each measurement was taken using a 51 s detection time with 256 scans, a spectral range of 4000-675 cm^-1^ and a resolution of 8 cm^-1^ and collected in triplicate. The detection limit of the instrument is 20 µm. When particles were smaller than 20 µm, MPs/NPs were grouped into a pile to be equal to or greater than 20 µm, ensuring all IR energy was directed at the MPs/NPs sample rather than background.

**Section 2.3.4. Metal Sorption to Particles.** Prior to measurement in ICP-OES, 1 mL of sample was digested for 24 h by adding trace metal grade HNO_3_ (Sigma-Aldrich, St. Louis, MO, USA) to achieve a concentration of 29% HNO_3_ in the solution. Subsequently, the solution was diluted using ultrapure water (16–18 mΩ, Barnstead GenPure Water, Thermo Fisher Scientific, Waltham, MA, USA) to obtain a final concentration of 5% HNO_3_. In accordance with previous studies (Lasheen et al. 2012), the PET filter was inverted and subjected to a 4 mL 0.2 M HCl desorption process in an orbital shaker at 200 rpm for 30 min to release the Cd retained in the filtered particles. Subsequently, the solution was diluted to 40% HCl with ultrapure water and analyzed trough ICP-OES. Standard reference material (NIST SRM 1643f; National Institute of Standards and Technology, MD, USA) was also analyzed to ensure the quality of the measurement. Recoveries > 90% were achieved.

**Bibliography**

Lasheen MR, Ammar NS, Ibrahim HS (2012) Adsorption/desorption of Cd (II), Cu (II) and Pb (II) using chemically modified orange peel: Equilibrium and kinetic studies. Solid State Sci 14:202–210
